# European Correspondence

**Published:** 1878-06

**Authors:** R. J. Nunn

**Affiliations:** London


### EUROPEAN CORRESPONDENCE.

LONDON, April 26, 1878.

*Editor Atlanta Medical and Surgical Journal:*

A winter passed among the continental universities leads me to enquire the motive which prompts so many American students to rush over the continent and rush home again with the idea of having studied at a foreign school. Is it profit, pleasure, or puff? Is it a fact that the scientific education offered to the American student (I do not speak of one school, but of all together) in the first-class American schools is inferior to that afforded on the continent? Now, I hazard the assertion that it is quite equal; I do not speak of the many minor medical schools scattered broadcast over the land—all useful, perhaps, in their way, but which lack the wealth necessary to acquire first-rate apparatus and appliances, and to pay first-class teachers or professors—but I speak of those larger schools, whose means are ample for all the professors required. The fact is that the education is offered, but as a general rule the students will not accept it. An American student graduates in two years. (The day is gone by, I hope, when it could be done in seven months, as I have known it to occur.) Will he then be satisfied to continue for three more years to occupy the student's bench, and make the term of his tutilage equal to that of his German brothers? One in a thousand might, but even that one is very doubtful. In Germany the student goes, if he wishes, from school to school, studying at each that particular subject which he finds may be best prepared to suit his mental appetite; and as he may learn physiology at one school, anatomy at a second, surgery at a third, practice at a fourth, etc., of course the sum total of the lectures must amount to the legal requirements, but the student is free to take them when he pleases, and he is not hampered with the regulation as with us, that a whole course of eight or ten branches, as the case may be, must be taken in a particular school, some of whose professors, although first-rate men in their way, might not suit, in their manner of teaching, the mental requirements of the student in question.

The evils of our system of medical education can be corrected; nay, they will, they must be corrected later, doubtless, by legislative enactments, but for the present we must trust to the good sense of the students themselves and to the moral influence of the profession to gradually awaken the coming generation to the necessity for higher educational requirements.

But it must not be imagined, because I thus criticise our own institutions, that our professors have not kept up pretty well with our attainments in the arts and sciences when compared with the old world. In this, the beginning of the second century of national existence, our system is in a much less chaotic condition than it was in England or on the Continent fifty years ago, with governments many centuries old.

There is yet another reason why the majority of our students can derive but little advantage from a short attendance at the continental universities, and that is the question of language. Of those going abroad, very, very few know enough of the language to understand a scientific lecture, although their knowledge may be ample for the requirements of travel. Indeed, during the past winter I have spoken with several American graduates, who frankly confessed that they could not follow the lectures, although in the practical branches they could get along very well. Yet if there is one thing more than another in which the genius of American medicine will excel, it will be in the more practical departments. Now, strange as it may appear, many of these men, after spending a few months on the continent, were prepared to return home, entirely ignoring and passing by the English school, where, if any place out of America, they might derive most advantage from their opportunities on account of the identity of language.

I think, then, that, with rare exceptions, it is not profit in the sense of educational advancement which the average American medical student derives from a winter on the Continent.

The novelty of the pleasures of a continental life has for a young man many allurements during the first few months of his residence there, and few can bring the same patient, quiet industry to bear on their studies which they would do at home; and about the time this novelty begins to wear off is about the time they turn their faces homeward—professionally, perhaps, rather worse than better for their foreign trip.

But now we come to the whole secret of the young man's trip abroad—puff. It is, in fact, "a good send-off." "He has spent a winter in Paris or Vienna" will make a long credit column in his professional account, no matter how that winter was occupied. The absence of medical schools in the American colonies drove the students to Europe for their education, and the necessities of the early colonists began the habit of their descendants and developed into the present day, when the real necessity has long since disappeared.

I have spoken of the real utility of the many smaller medical schools scattered over the American continent, and here I would indicate what I believe is their true use. Very many of these schools cannot pretend to offer to the student a scientific education of the same high order as in the larger ones. If Prof. Ludwig requires a lecture every day for a whole winter and a thousand vivisections to teach physiology, it cannot be supposed for a moment that the same can be done in one-fourth the number of lectures and a dozen experiments. But no matter when or how, let the student but have a thorough scientific knowledge, and then a session passed in the medical school nearest to the scene of his future labor—not farthest from it, as at present—will be the best possible introduction to the practical duties of his profession. A student expecting to practice among malarial districts may expect to meet with many perplexities if he has studied only among typhoid diseases. Again, the habits of the people among whom he is to practice are of great importance, and these can be best studied in local medical schools.

May I say that this is no ideal picture, but the record of facts which have come under my own observation? May I then record as the opinion of (I hope) a candid and unbiassed person, that America can educate physicians for her own people better than foreign nations can? Of surgery I say nothing, as, being more mechanical, much depends on the ingenuity and boldness of the individual, and here I think America is well in the foreground.

It must not be thought, however, that I do not appreciate fully the advantages to be derived from a visit to foreign schools, but who are the men who will derive most advantages from such visit? I take it to be the men who are already masters in their business, or who, having been forced or drifted into practice of a specialty, are able to compare analyses and estimate the value of their observations abroad.

All this may appear out of place, but it comes from a desire that the American profession should learn to estimate things at their proper value, and cease to worship at foreign shrines when they have scientific altars quite as magnificent at their own doors.

In all the large European cities the same classes of diseases appear to prevail, and but for the differences of race and language, a physician could easily mistake a London clinic for one at Paris, Berlin or Vienna, or *vice versa*,; and if I include our large Northern cities, I will not be much mistaken.

Insufficient ventilation, scanty food, darkness, dampness and over-crowding produce the same results in one place as in another, when climatic influences are similar, and there is not sufficient topographic or atmospheric differences between these several capitals to make a visible change in the constituent elements of a clinic, although exact statistics might show a considerable distinction. Nor will so much novelty of treatment, *reliable treatment*, be met with as one might suppose. In the present advanced state of medical journalism medical discoveries become common property almost as quickly as steam can carry them throughout the world.

With many electricians I find the Daniel's battery still in high favor for office purposes, where size and weight are not matters of vital importance, and many modifications are in use to get rid of its inconveniencies, but I think the most convenient I have seen is that in which the negative plate and crystal being at the bottom of a jar, a thick layer of wet clay is put on them and then the zinc plate. The use of the clay is, I understand, original with Prof. Hughes, the inventor of the microphone, which magnifies all sounds transmissable by the telephone, so that sounds otherwise inaudible become startlingly loud.

The microphone has been constructed by Prof. Hughes of various substances and in many forms, but the substance he at present prefers is charcoal impregnated with metal, as for example, willow charcoal, which has been heated in a closed sheet iron tube to a white heat and allowed to cool slowly, or gas carbon heated to redness in a closed crucible and then plunged into cold mercury. Both of these are employed by the inventor for different forms of the apparatus, but the most portable form being made with the ferrated charcoal, I will confine my remarks to that.

Make three or four little bricks of this ferrated charcoal about 3-16 or ¼ inch square and about ½ inch long, pile the bricks one upon the other; attach to the upper and lower brick, wires for completing a galvanic circuit, and the apparatus is complete—the rest of the arrangement being only for the purpose of maintaining these pieces of charcoal in their relative position certainly, but not rigidly. For this purpose the upper and lower bricks are cemented to two bits of wood, which are hinged together, while the intervening pieces are attached to the lower one by a strip of paper gummed to one side. A gentle spring draws the pieces of wood together and insures contact between the pieces of charcoal. This is the sender or transmitter. The receiver is a Bell's telephone, which is connected with the sender by one small wire. A small galvanic battery is placed in connection with the other wire of the sender and the second terminal of the telephone. The strength of this battery is governed by the magnetic strength of the telephone, from 3 to 6 Daniel's cells, exposing about two square inches of zinc each, will usually be sufficient. A galvanometer may be placed in the circuit for the purpose of inspection if desired. It appears that a number of senders and receivers may be connected in the same line and a variety of sounds sent to and fro without interfering one with the other.

In using this arrangement the sender is placed so as to receive the sound to be transmitted, which instantly becomes audible at the receiver (telephone). For example, if placed on a table and the table scratched or tapped lightly, or even brushed lightly with a feather or camel's hair pencil, the sound can be distinctly heard at the other end of the line. Even a fly walking in a match-box with which the sender was in contact could be plainly heard. In the same way a whispered conversation can be carried on, but for this purpose the inventor prefers to enclose the receiver in a little box, so as to cut off, as much as possible, disturbing noises.

I have spoken of "sounds transmitted by the telephone." I have been experimenting a good deal with the telephone in consultation, and although I have no doubt there is a great future before this system of causing waves of sound to produce changes of electric currents, still, as a caution to enthusiasts, I would mention that there are *whole classes of sounds* to which it is *absolutely insensitive*, even when aided by the *microphone.* In other words, no arrangement has yet been found equaling in sensitiveness the human ear. For example, hissing and blowing sounds produce no deflection of the needle of the galvanometer; nor could I find any effect produced by the friction of mucous membranes, as, for instance, the rubbing of the tongue against the roof of the mouth. This may be well illustrated by pronouncing into the instrument the letters of the alphabet, when it will be found that r causes the greatest deflection of the needle; then ah, i, o, u, etc.—in fact, those sounds which are the results of vibrations—while consonants, as a rule, are only heard by the vowels associated with them, so that if a consonant is disassociated from its accompanying vowel it is not transmitted by the microphone or telephone; nor will it be recorded by the phonograph. In other words, such sounds will not excite vibrations in any of the substances at present employed for the purposes of transmission or record. Hence, as an example, if a prolonged s, or f, or v, etc., is pronounced into the microphone no deflection of the needle takes place for the latter or consonental part of the sound. It will be seen, therefore, that further improvements must be made before these instruments become available as reliable aid to the auscultator. I hope I have succeeded in making my views on this subject understood. I have not aimed at scientific accuracy, but only to place my observations broadly before my medical brethren.

While on this subject, I would like to draw attention to the value of antimony as a substitute for carbon in certain forms of galvanic batteries; those, for example, in which sulphuric acid or chromic acid is the exciting fluid. I have used it, I think, about five years. It has the advantage of being cheap. The broken plates can be recast. It does not disintegrate, or flake off. Moreover, when plunged into the fluid it commences to act immediately. Although perhaps not quite so good a negative as carbon, its superior conductivity and other advantages may make it much more convenient for the medical electrician. Its great disadvantage is the brittleness of the plates; but this I have corrected by either casting it on a core of tough metal, as copper plates, or by slightly alloying the antimony itself.

In gynæcology I can recall nothing startlingly novel that I saw. In spite of the hundreds of specula invented, I find the Ferguson maintaining its place as a clinical instrument; and the number of pessaries about equals the number of gynæcologists, each one being conscientiously bound to invent a pessary for himself, which he can praise over all others. In fact, the judicious selection and application of the proper pessary to suit each case in which one is required at all, is perhaps one of the most difficult and unsatifactory subjects with which the clinical gynæcologist has to deal. In engorgements of the os I find puncturing a very favorite method of local depletion, taking the place of leeches, both artificial and natural, as requiring less time, but I cannot say that it seems to me so effectual. In obstinate induration of the os the use of caustic potass seems to be in favor, but its employment requires great caution on account of the danger of serious inflammation following its application. The treatment of cancer by the subcutaneous injection of bromine is recommended by Dr. Williams, but from what I can see it also is only a more desperate remedy for a fatal disease.

For some time I have been investigating, as opportunity offered, the very remarkable powers of suction, retention and expulsion, voluntary and involuntary, possessed by the vaginal walls. In particular, the value of the voluntary muscular efforts of the parturient female is recognized, but such, I think, is not the case with similar powers possessed by these organs in their normal condition, or, if it has been, it has fallen into obscurity, and is not given that prominence as a remedial agent in cases of displacement of the uterus to which I consider it to be justly entitled.

The degree of force and of capacity varies in different individuals. The former is rather difficult to estimate, requiring the construction of special instruments, which I have not yet been able to perfect. At present, therefore, I can do little more than state the fact. The greatest capacity or power of retention of fluid (water) which has come to my knowledge is half a pint, and this case I obtained through Dr. H. E. Gantillon, Paris, who has kindly consented to aid me in these investigations. Gynæcologists can easily investigate this subject for themselves, but they will find many women who do not possess this power, or, rather, who do not know how to exert it. They must, therefore, be prepared to exercise a little patience in their research, for like all other voluntary muscular action, it can be increased by practice and ultimately become, not involuntary, but unconscious muscular action.

To me it appears to be a mistake to look upon the uterus as wholly an inert organ, subject only to the laws of gravitation, etc., to be pushed about at will, and supported by mechanical contrivances alone, without consulting the voluntary efforts of patient to aid in its support, or without instructing her how to utilize those voluntary muscular efforts to assist in replacing a displaced uterus; and, again, it is highly important that she should be informed how to avoid those expulsive movements which would aggravate her condition if not absolutely cause it.

This remarkable power, when applied to the inhalation and exhalation of air, *as I have seen it*, offers an explanation of many phenomena, such as the crying of a child "in utero," etc., and might give rise to some curious questions in jurisprudence.

It is by no means my intention to propose this as a means intended to supersede the several treatments at present employed, but to suggest it as a most important adjunct in this class of troublesome diseases, whose treatment is on the whole most unsatisfactory. Many displacements I conceive to be more or less the result of a vicious habit, like round shoulders or stoops, and to be capable of amelioration by similar means.

Syphilis, notwithstanding all that has been written about it, appears still to be combated by the same remedies with which we are already acquainted. In the clinic of Prof. Auspitz, at Vienna, I saw the expectant treatment adopted for chancre of every variety, internal remedies only being given where constitutional symptoms appeared. This is entirely opposed to my judgment. For years it has been my custom to treat all cases of syphilis as if constitutional, and then I know I am sure not to err. I know the objections which may be urged against this course, but I think these will be far outweighed by its advantages.

Chronic urethral inflammations are principally treated in the clinic of Dr. Ullzman with injections of one or two per cent. solution of alum or tannin, or both, administered through the catheter.

A magnifying ophthalmoscope has just been invented by Dr. Englehardt, of Munich. In this instrument, refraction is used instead of reflection. The light from a lamp falls on a lens, which causes the light to fall at the angle of polarization on a piece of plate-glass. The light refracted from this plate falls on a second lens, and, concentrated by it, enters the eye to be examined. The examiner, seated in the usual position, sees the image of the eye through the plate-glass, and a lens or two magnify it to any required size. This arrangement, with the necessary boxing, draw-tubes, etc., constitutes the finished apparatus, but a plate of glass to refract the light and a lens next to the patient's eye to magnify the image makes a practical and useful instrument, as the inventor took much pains to demonstrate to my satisfaction. The same gentleman has invented and uses a cataract forceps, for the positive extraction of cataract.

I do not know if "Drypsine" has yet reached you. It is a principle similar to pepsine, discovered in the pancreas. It has the curious property of dissolving all animal tissues except cellular tissue. I am not acquainted with the method of its preparation, but it does not seem likely to be of much medical importance, as pepsine with pancreatine accomplish the same results for medical purposes.

I desire to call attention to a method of mapping out, as it were, the constant changes of the vital elements (if I may so call them), respiration, pulse and temperature, going on in the system. By assuming for each of these an average of health, say R 18, T 98, P 76, and adding together, a sum is obtained, 192, which may be called a vital number in its simplest form; but I have found it useful to make the picture more striking, and for this purpose I have added the elements in various combinations, thus: R 18, P 76, R+P=94, T 98, R+T=116, P+T=174, R+P+T=192. To obtain a graphic representation of these numbers, I assume some unit to be some length, say one-twentieth of an inch, and drawing a line across a piece of paper, I cross it with seven perpendicular lines half an inch apart, one for each of the elements to be expressed, and call them R, P, etc., respectively. From the cross line I measure downwards on the R line 18-20 of an inch, on the P line 76-20, and so on through the other lines; and by drawing a line through these measurements, I obtain a curve which might be called a standard or health curve; but by assuming to reckon from this curve upwards, the cross line becomes the standard or health line, and this is the line I have used as such, because it is more easily remembered than any other, and hence variations can be compared with more facility.

To use this system, variations above or below the health line are to be measured in twentieths of an inch from the health line, and the points so obtained being joined by a line, give a curve of variation above or below, as the case may be. Thus, R 24 is 6-20 above, P 100 is 24-20 above, etc., R 12 is 6-20 below, P 50 is 24-20 below, etc.

In practice I count the T by tenths of a degree for units, and I use 1-100 of an inch as the unit of length, and the system might be extended to the extent of becoming unwieldy. In its present form, although difficult to describe, it will be found exceedingly simple and very graphic, bringing the daily changes of a patient before the eye with great vividness.

These vital curves (if I may be permitted the name) only supplement the tables of temperature, etc., at present in use, and I have so arranged the forms that both series of lines can be carried on the same table. I have also adopted the system of colors to distinguish the elements from each other, which I find adds somewhat to its distinctness.

I have trusted to this clumsy method of description in preference to sending you a table, because I feared the printing of the latter might be inconvenient to you; but if you desire, I will feel pleasure in sending you one, although you could make one yourself in one-eighth the time it has taken to describe it.

My attention has been directed to some beautiful stereoscopic pictures of anatomical, surgical and pathological specimens, and it is rather curious that these have not become more common, since they can be so cheaply multiplied by printing by the autotype, the Woodbury, or the German Lichtdruck process; and further, there is now no reason why these or other prints of that character should be issued uncolored, for by a recent discovery, made by Mr. Albert, of Munich, this can be affected easily without the aid of an artist. Mr. Albert simply takes three negatives, one each through red, yellow and blue glass; from each of these a Lichtdruck is made and printed with ink of its respective color. The print is made from these three plates, as in chromo lithographing, in an ordinary lithographic press, and the result is is a very satisfactory colored print. When coloring is of so much importance, as in medical works, and this can be so easily and cheaply obtained, there can be but little excuse for issuing books with uncolored illustrations.

R. J. Nunn, M.D.

